# Influence of Specific Heat Input and Weld Configuration on Hardness and Residual Stress Distribution of S960MC Steel Welds

**DOI:** 10.3390/ma19102062

**Published:** 2026-05-14

**Authors:** Matus Murin, Libor Trsko, Frantisek Novy, Martin Fratrik, Michal Jambor, Vratislav Mares

**Affiliations:** 1 Faculty of Electrical Engineering and Information Technology, University of Zilina, Univerzitna 8215/1, 010 26 Zilina, Slovakia; matus.murin@feit.uniza.sk; 2 Research Centre UNIZA, University of Zilina, Univerzitna 8215/1, 010 26 Zilina, Slovakia; 3 CPIT—Centre for Advanced Innovation Technologies, VSB-TU Ostrava, 17. listopadu 2172/15, 708 00 Ostrava, Czech Republic; vratislav.mares@vsb.cz; 4 Faculty of Mechanical Engineering, University of Zilina, Univerzitna 8215/1, 010 26 Zilina, Slovakia; frantisek.novy@fstroj.uniza.sk (F.N.); martin.fratrik@fstroj.uniza.sk (M.F.); 5 Institute of Physics of Materials, Czech Academy of Sciences, Zizkova 22, 616 00 Brno, Czech Republic; jambor@ipm.cz

**Keywords:** welding, high strength steel, S960MC, residual stress, X-ray diffraction

## Abstract

This study investigates the influence of specific heat input and weld configuration on heat affected zone hardness and residual stress of S960MC high strength steel welds. In total, five types of weld samples were manufactured by Tungsten Inert Gas (TIG) autogenous welding and Metal Active Gas (MAG) butt welding to simulate the effect of increasing heat input and constraining the relative motion of welded parts during the heating and cooling phase. The obtained results show that the highest axial tensile residual stresses with magnitude above 900 MPa, combined with a hardness drop in a range from 13 up to 18%, occur mostly in the sub-critical heat affected zone, making it the critical zone of the weld. Increasing the heat input during welding does not have a simple correlation with generating more residual stresses and the trends obtained on the surface are different from results evaluated at a depth of 0.2 mm. Restraining the relative part motion during the welding affects mostly the tangential residual stresses, causing an increase in their tensile magnitude localized in the middle of the heat-affected zone while almost no influence on the axial residual stress component was recorded.

## 1. Introduction

High-strength low-alloy (HSLA) steels form an individual material group which gained significant importance in industry due to a favorable weight/strength ratio and high mechanical properties [1]. Their most significant advantage is that their unique mechanical properties are not achieved by increasing carbon content and rather by multiple strengthening mechanisms, such as grain refinement, dislocation strengthening, precipitation strengthening and phase transformation of austenite to martensite or bainite [2,3,4]. Thanks to the low content of alloying elements, these steels have a favorable carbon equivalent value and are suitable for welding with all conventional techniques [5,6,7].

On the other hand, as the applied strengthening mechanisms are naturally temperature unstable, welding of the HSLA steels has multiple specific negative aspects that need to be understood and considered in the welding process. The most challenging aspects of HSLA welding include susceptibility to cold cracking in the heat affected zone (HAZ) or weld metal (WM), softening in the HAZ and a significant decrease in toughness and ductility in the weld area. Due to the high temperature microstructure recovery processes, the most frequently observed negative aspect is a decrease in mechanical properties in the HAZ to a level below the mechanical properties of the material in as-received condition guaranteed by the manufacturer. These aspects have slowly unfolded thanks to multiple systematic studies performed in recent years such as those in references [8,9,10,11]. An important study was carried out by Amraei et al. [12], where the authors evaluated the microhardness of S960 HSLA steel samples heated to temperatures representing individual subzones of the HAZ and using of correlation equations between hardness and ultimate tensile strength (UTS) and determined theoretical strength. The results showed a 32% UTS drop when compared to the as-received material. In a second study by the same authors [13], it is concluded that the hardness drop in the HAZ of S960 steel can reach up to 46%. Generally, it is well understood that the key parameter influencing the negative aspects of HSLA welding is the size of specific heat input, and that the most critical sub-zone is the one heated below the Ac_1_ temperature. Heating causes recovery of the thermomechanically processed microstructure, mostly by decreasing the dislocation density accompanied by releasing the strain hardening [8,14,15].

One of the HSLA welding aspects still not fully understood is generation and distribution of residual stresses, which are created primarily due to thermal expansion and shrinkage of the melted and heated material and secondarily due to the phase transformations from the liquid to solid state and within the solid state (polymorphic metals) [6,16]. This aspect gains increasing importance as it was recently proven by Tichon et al. [17] that residual stresses are often the determining factor for fatigue crack propagation and might be more important than microstructural variation. The magnitude and distribution of residual stresses generated by welding depend on several factors, while the most significant still appears to be the specific heat input. Among other relevant factors that can be considered, the used welding technique, welding sequence, pre-heating, post-heating, type of filler metal and the total weld’s length should also be considered [7,18,19,20,21]. Alipooramirabad et al. [22] compared the influence of used filler metal and different welding sequences on residual stresses of API 5L grade X70 steel multi layered welds. The results have shown that higher residual stresses are generated by the flux cored arc welding process compared to conventional shielded metal arc welding and modified short arc welding. The highest magnitude of residual stress was obtained in the weld metal, and in many cases, it exceeded the yield point of the base metal. The authors also conclude that significantly higher residual stress magnitudes were obtained in the longitudinal direction when compared to the transverse and normal direction. Interestingly, the results also provided the analysis of the residual stress profile along the thickness of the weld, while the highest magnitudes were observed near the surface and decreased towards the root of the weld. This was caused by the fact that the heat generated during deposition of the subsequent passes imparts a tempering effect on the previous passes. In another work, Alipooramirabad et al. [23] analyzed the influence of specific heat input on residual stresses of multi-sequence welds while the heat input was modulated via welding speed. When using higher welding speeds, the specific heat input was 7.7 kJ/cm, while at lower speeds the heat input reached value of 19.1 kJ/cm. The analysis showed that, unexpectedly, slightly lower residual stresses were obtained at higher heat inputs. This was most likely related to different cooling rates what led to different phase composition of the microstructure in the weld metal and HAZ. In addition, the higher heat input had a higher tempering effect on the previous passes. Schroepfer et al. [24] studied how the filler metal strength and heat input value influences the size of reaction forces created during the material shrinking and how it is related to the post-weld residual stresses of S960Q HSLA welds. The authors concluded that when using a low heat input, the high strength G89 filler metal leads to decreased stress levels compared to the lower strength filler metal G69 due to effects of phase transformation. Based on the results, it was stated that the heat input for over-matching filler materials must be lower than for lower strength steel grades to maintain optimized residual stress levels.

In general, residual stress generation in welded joints is a highly complex task, and published experimental results show high ambiguity and are often even contractionary. For instance, in the work of Guo et al. [25] an experimental analysis was performed on S700 steel showing peak axial tensile residual stress magnitudes in the middle of the HAZ and lacking any information about tangential stress. In contrast, in the work of Sisodia et al. [26], where residual stresses on S960 steel were analyzed, results show highest axial tensile residual stresses on the boundary between the WM and the HAZ, while in the tangential direction in the whole WM and HAZ, only compressive stresses were recorded. In addition, the majority of the related works [16,26,27,28,29,30,31] focus only on the near-surface residual stresses omitting the possible in-depth stress transformation due to the self-equilibrating nature of residual stresses and significant influence of the weld configuration (available degrees of freedom for relative motion of the welded components). Therefore, to provide more comprehensive information about the residual stress development in HSLA welds, this study focuses on systematic mapping of residual stresses in five different welds on the surface and at a depth of 0.2 mm, evaluating the influence of weld technology, component restraining and variations in the heat input. The evaluation was performed at the beginning of the weld, where the components are initially at room temperature, in the middle and at the end of the welded joint, where the components are hottest due to the generated heat from the electric arc.

## 2. Experimental Material and Preparation of the Weld Samples

**Figure 1:**
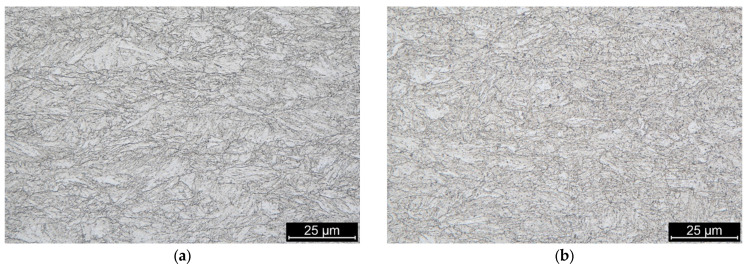
Microstructure of the S960MC sheet metal in axial (**a**) and tangential (**b**) orientation with respect to the rolling direction.

The experimental analysis was performed on S960MC HSLA steel in the as-received condition and with chemical composition shown in Table 1. It is a thermomechanically processed steel used for welding and cold forming. The steel contains Nb, V and Ti microalloying elements to form precipitates, which tend to block the recrystallization processes to maintain ultra-fine-grained microstructure after thermomechanical processing. Mechanical properties of the used 4 mm thick sheet metal obtained by a conventional tensile test are provided in Table 2. Microstructure of the experimental material revealed by optical microscopy (Figure 1) contains bainite, tempered martensite, retained austenite and precipitates (carbides of Nb, V and Ti).

**Figure 2:**
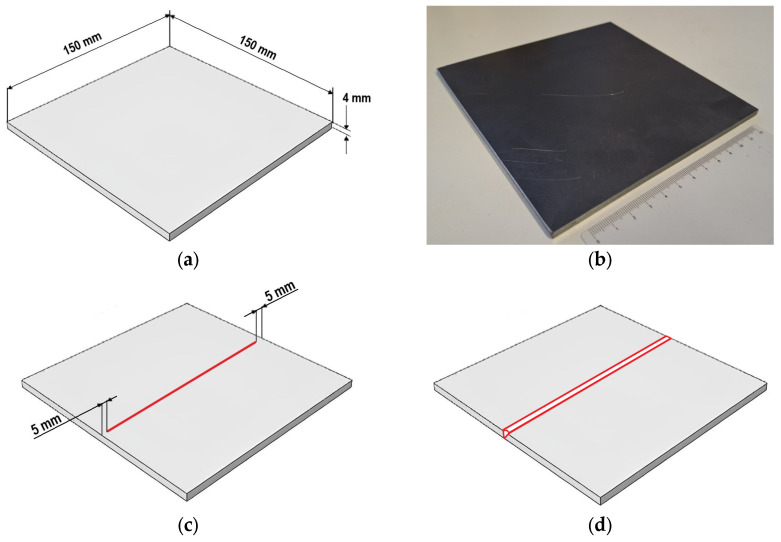
Experimental samples and process of welding: (**a**) shape and geometry of the samples; (**b**) the real manufactured sample; (**c**) location of the autogenous line weld joint on Samples 1–3; and (**d**) location of the butt weld joint on Samples 4–5. The red lines designate the weld location.

To manufacture the weld specimens, a 4 mm thick S960MC sheet metal was sectioned to dimensions of 150 × 150 mm, as shown in Figure 2a,b. For the first three samples (designated as Samples 1–3) a Tungsten Inert Gas (TIG) autogenous weld was performed in the middle of the sectioned sheet metal starting and ending 5 mm from the edge (Figure 2c). The process was essentially just remelting the material with use of a TIG welding torch to simulate a weld configuration, when the joined components are fixed together and unable to move freely during the heating and cooling. Additionally, on Sample 3 a root filling weld was simulated by re-melting of the material from the opposite side (bottom) of the sheet metal. The sheet metals for Samples 4 and 5 were sectioned in the middle to create a typical welding configuration, when the components are completely separated and can move freely during the welding. Afterwards, Sample 4 was TIG autogenously welded without use of filler metal. Sample 5 was welded by a conventional Metal Active Gas (MAG) welding process with use of G3Si1(G 42 4 C1/M21 3Si1, Voestalpine, Linz, Austria) wire filler metal (dia. 1 mm), as shown in Figure 2d. The welding process was always carried out at room temperature, and in the case of Sample 3, the material was fully cooled down prior to the secondary root filling.

To eliminate the influence of a human operator, all experimental samples were welded with use of automatic welding robot. The samples where the TIG method was used (Samples 1–4) were welded by FANUC LR Mate 200iD4S robot (FANUC, Oshino, Jamanasi, Japan). using Fronius MagicWave 2200 as the welding source. For the MAG weld of Sample 5 a KUKA VKR 250/2 robot (KUKA, Augsburg, Germany) was used with Fronius TPS 4000 CMT (Fronius, Wels, Austria) welding source. The full welding process parameters for all specimens are given in Table 3. The specific heat input Q was calculated by the following equation:

(1) $$Q\;=\;\frac{U_{W}\times I_{W}}{V_{W}}\times \;\eta \;[J/\mathrm{cm}],$$

where U_W_ is the welding voltage [V], I_W_ is the welding electric current [A], V_W_ is the welding speed [cm/s] and η is the heat transfer coefficient (for TIG η = 0.6 and for MAG/MIG η = 0.85).

The welding parameters for Sample 1 were experimentally obtained as the lowest heat input, which caused visible heating mark on the bottom of the sheet metal, proving that the material was thermally affected through the whole thickness. The heat input for Sample 2 was chosen as a 25% increase compared to Sample 1 to achieve a notable difference in the heat input and avoid unwanted microstructural changes and the creation of welding defects. Sample 3 was welded with the exact parameters as Sample 2, but additional remelting of the weld root was performed. For Sample 4, the welding parameters were kept very close to Sample 2 with minor modifications respecting the presence of a welding gap. The MAG weld parameters used for Sample 5 were chosen to achieve full weld penetration. The other related parameters of the thermal cycle for identical material with similar heat inputs was extensively studied in [9] and serve as a baseline for the following experimental analysis.

## 3. Experimental Analysis and Obtained Results

### 3.1. Residual Stress Analysis

#### 3.1.1. Residual Stress Analysis Methodology

**Figure 3:**
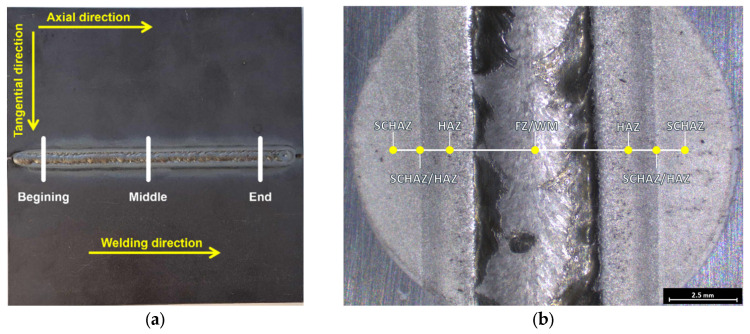
Scheme of the residual stress analysis positions on welded samples: (**a**) scheme of the residual stress analysis locations concerning the weld joint and (**b**) the scheme of residual stress analysis locations concerning the HAZ.

For residual stress evaluation, a sin^2^Ψ X-ray diffraction technique was applied. The used device was a Proto iXRD Combo with process parameters as follows: CrKα radiation, a total of seven tilt angles in the range of ±30°, and a Bragg angle of 156° (given by diffraction of {211} planes), with elastic constants for calculation of ferritic steel’s residual stresses: ½s_2_ = 5.75 TPa^−1^ and s_1_ = −1.25 TPa^−1^; irradiated area of 1 mm^2^. The measurement locations with respect to the heat affected subzones are designated in Figure 3. 

**Figure 4:**
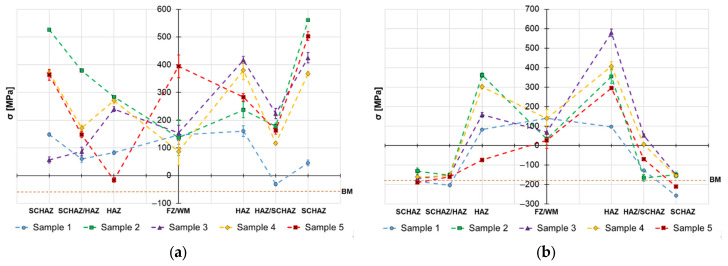
Welding residual stresses–beginning of the weld joints–surface: (**a**) axial direction and (**b**) tangential direction.

**Figure 5:**
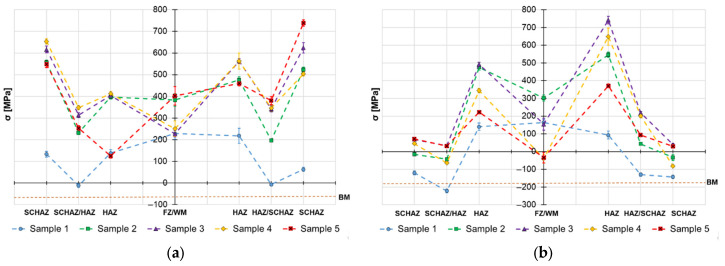
Welding residual stresses–middle of the weld joints–surface: (**a**) axial direction and (**b**) tangential direction.

**Figure 6:**
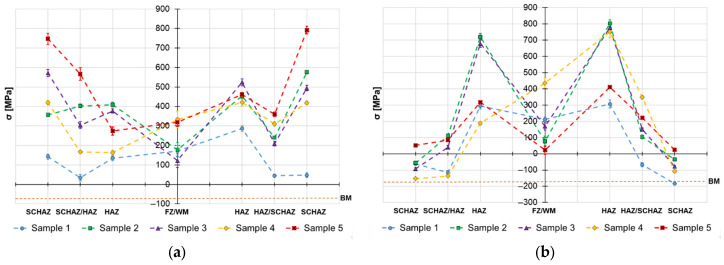
Welding residual stresses–end of the weld joints–surface: (**a**) axial direction and (**b**) tangential direction.

**Figure 7:**
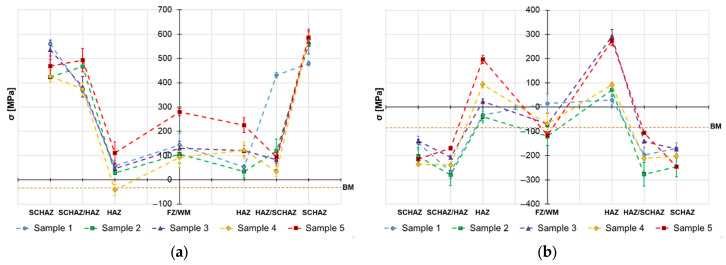
Welding residual stresses–beginning of the weld joints–a depth 0.2 mm: (**a**) axial direction and (**b**) tangential direction.

**Figure 8:**
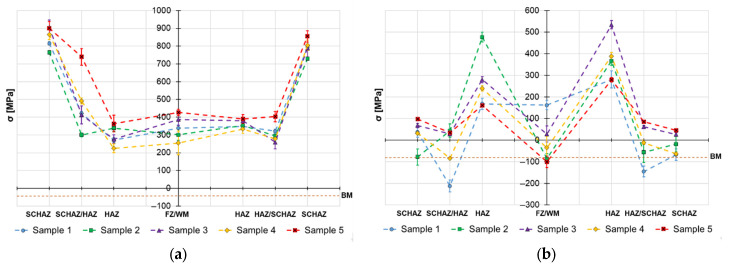
Welding residual stresses–middle of the weld joints–a depth 0.2 mm: (**a**) axial direction and (**b**) tangential direction.

**Figure 9:**
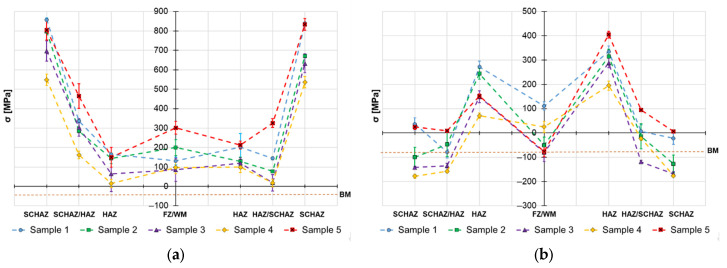
Welding residual stresses–end of the weld joints–a depth 0.2 mm: (**a**) axial direction and (**b**) tangential direction.

The sin^2^Ψ X-ray technique is designed to evaluate the residual stress from several measurements performed in one location at different inclinations (tilt angles) and extrapolate the results to the desired φ–angle, which defines the stress orientation (e.g., axial, tangential). The advantage of this method is that each measurement provides statistics based on multiple measurements, together with a standard deviation value (plotted in all the residual stress graphs in Figure 4, Figure 5, Figure 6, Figure 7, Figure 8 and Figure 9 as error bars with values typically lying between 3 and 10% of the measured stress value). The irradiated area is defined by the diameter of the X-ray beam collimator, which is the factor determining the lateral resolution of the measurement technique. In this case, a collimator of 1.13 mm diameter was used, therefore to avoid overlapping of the irradiated area, the distance between adjacent measurement locations must be at least equal to the collimator diameter.

The residual stress analysis of the welded joints was performed on the top side of the weld by discrete line measurement in a direction perpendicular to the weld axis in the beginning, middle and end of the weld in axial and tangential directions, as depicted in Figure 3a. The bottom of the weld (root side) was not investigated. The surface oxide present on the sheet metal and on the weld had to be removed prior to the measurement by electro-chemical polishing to a depth of 0.03 mm to reveal the clean material surface. The polishing was performed with use of saturated water solution of NaCl as the electrolyte at 24 V and 2 A. After the removal, the HAZ was roughly visible and with respect to the lateral resolution, the line measurements were performed in seven locations, as shown in Figure 3b. After measuring the surface residual stresses, a second electropolishing was performed to reach a depth of 0.2 mm, and another set of residual stresses measurements was carried out. The reference value of residual stresses in the as-received material’s condition was measured on separate piece of sheet metal that was not subjected to any welding heat influence. It is also important to note that since in the TIG autogenous welds no filler metal is added (Samples 1–4), the weld mark will be referred as “Fusion Zone (FZ)” and in case of added filler material (Sample 5) it will be referred as “Weld Metal (WM)”. All the other HAZ subzones will be designated according to [9], as follows:

(a) Coarse-Grained Heat Affected Zone (CGHAZ): characterized by significant grain coarsening due to heating significantly above the Ac_3_ temperature;
(b) Fine-Grained Heat Affected Zone (FGHAZ): characterized by finer microstructure caused by heating to temperatures, which correspond to normalizing heat treatment;
(c) Inter-Critical Heat Affected Zone (ICHAZ): a partially recrystallized zone, due to heating between the Ac_1_ and Ac_3_ temperatures, where only partial austenitization occurs;
(d) Sub-Critical Heat Affected Zone (SCHAZ): area heated to temperatures lower than Ac_1_ corresponding to soft annealing.

#### 3.1.2. Results of the Residual Stress Analysis

The reference residual stress values of the base metal are provided in Table 4, showing that the surface and in-depth axial residual stresses are both of low value and can be considered as near-zero. However, in the case of the tangential direction, the surface residual stress exhibits magnitude of −183 MPa, which changes to −93 MPa at a depth of 0.2 mm. Even when these values can be still considered low, they are not negligible as in the case of axial stress.

The obtained surface residual stress data are provided in Figure 4, Figure 5 and Figure 6 and the in-depth residual stresses are plotted in Figure 7, Figure 8 and Figure 9. All the originally measured residual stress values, which were used for the residual stress plots (Figure 4, Figure 5, Figure 6, Figure 7, Figure 8 and Figure 9), are provided in Appendix A. Generally, the highest axial tensile residual stresses in both surface and in-depth measurements were recorded in the SCHAZ zone (Figure 3b). The highest magnitude of an axial tensile residual stress value in the SCHAZ zone was reached in Sample 3 (906 MPa), followed by Sample 5, exhibiting a slightly lower magnitude of 901 MPa. Other than that, Sample 5 reached the highest axial residual stress values in almost every evaluated location, making it the sample with the highest overall residual stress content. In the tangential direction, for both surface and in-depth measurements, all samples exhibit highest value of residual stresses in the middle of the HAZ. The highest recorded value was for Sample 2 (804 MPa), followed by Sample 3 (777 MPa).

The residual stress evaluation position in the beginning, middle and end of the weld showed that generally the lowest residual stress values were obtained at the beginning of the weld, where the material is initially at room temperature. Residual stresses have a tendency to increase in the middle location, while the effect is more pronounced in the axial measurement direction in both surface and in-depth measurements. At the end of the weld, the obtained residual stress values are slightly lower than the ones measured at the middle, indicating possible influence of the measurement position near the edge of the material. However, this might be related just to the actual length of the welded samples (150 mm), and shorter or longer welds may behave differently.

### 3.2. Hardness Evaluation

#### 3.2.1. Hardness Evaluation Methodology

To evaluate the mechanical properties of the weld’s subzones, a hardness measurement was performed using the HV0.5 method, following the guidance of the ISO 6507-1:2023 standard [32]. A line measurement of 60 indentation was carried out at a depth of 1 mm under the original surface, to be close to the location of the residual stress analysis, while still maintaining the required minimal distance from the surface required by the standard.

#### 3.2.2. Results of the Hardness Evaluation

**Figure 10:**
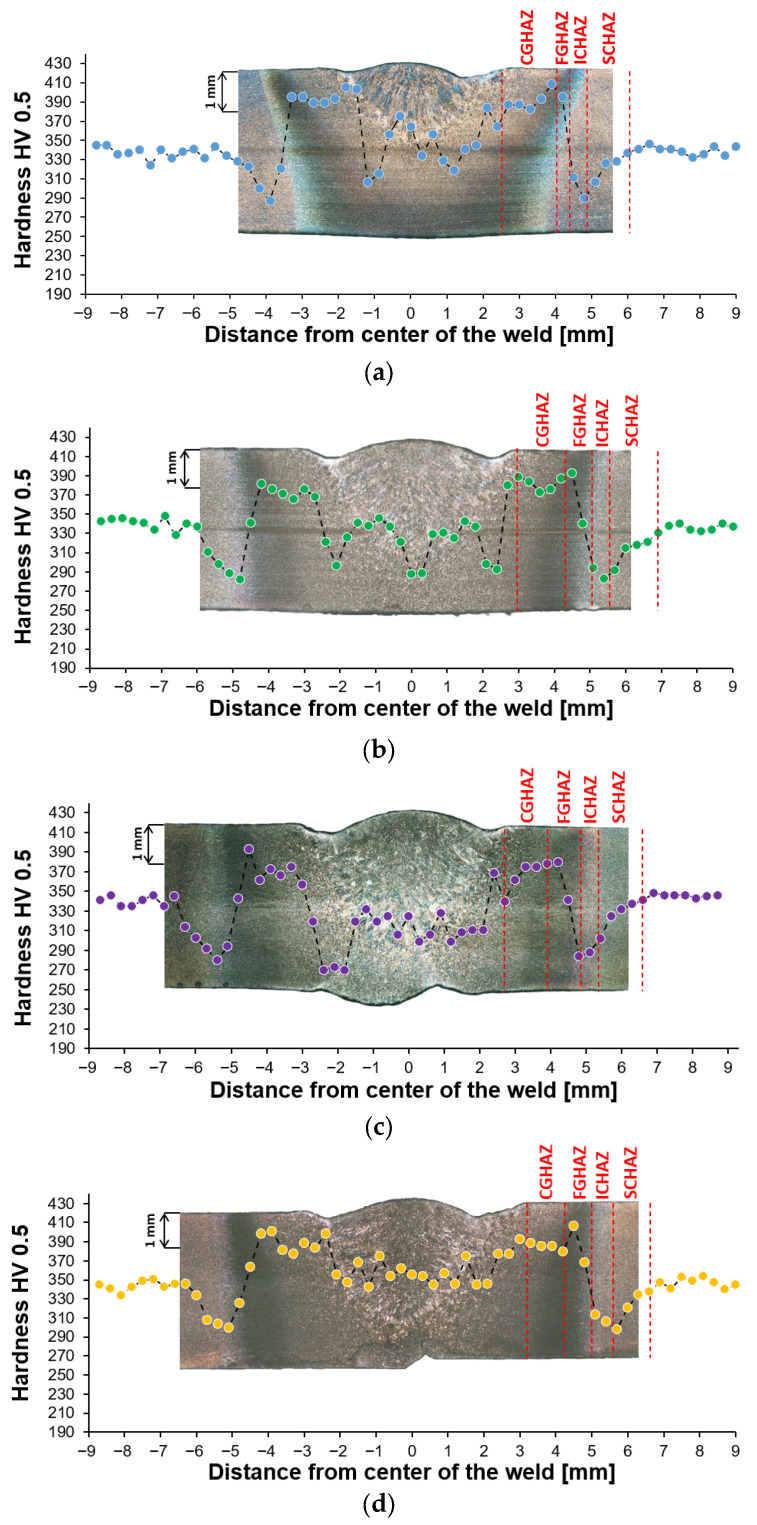

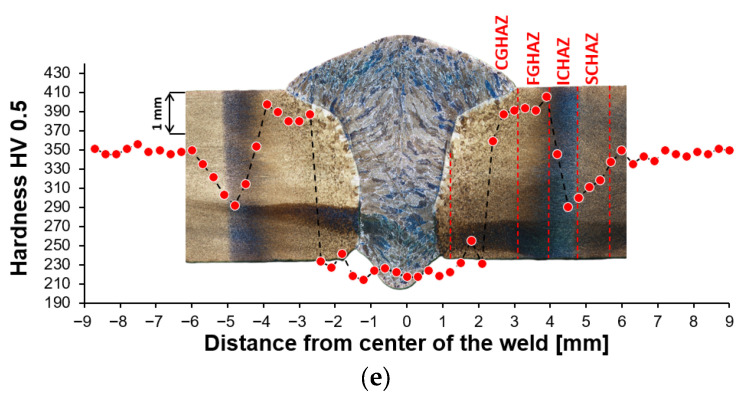
Hardness traverse measurement through the welded joint: (**a**) Sample 1; (**b**) Sample 2; (**c**) Sample 3; (**d**) Sample 4; and (**e**) Sample 5. The color dots represent the hardness measured in the specific weld location and dashed lines designate the approximate boundary between the individual heat affected subzones.

The microhardness traverse results for all samples are provided in Figure 10, and each measurement is paired with a metallography transversal macro-view for emphasizing the hardness values with respect to the individual subzones. The average hardness of the S960MC base metal in the as-received condition is approx. 340 HV0.5. In the case of the TIG autogenous welds (Samples 1–4), where no filler metal was added, the average hardness of the remelted material was very close to the hardness of the as-received material; however, the results are characterized by a high scatter (Figure 10a–d). In the weld toe, the hardness was approximately 40 HV0.5 higher with respect to the base metal. Further on, the lowest hardness values were recorded typically near the boundary between the ICHAZ or SCHAZ subzones of approximately 4 to 6 mm from the weld’s axis for all samples except Sample 3.

Sample 1 exhibited a decrease in hardness of the SCHAZ zone to approximately 290 HV0.5 (Figure 10a). Using a higher heat input for Sample 2 caused a decrease in the hardness for another 10 HV0.5 points to an approx. value of 280 HV0.5 (Figure 10b). A specific case was Sample 3, where the lowest hardness was observed in the location of −2 mm from the weld center in the CGHAZ zone (Figure 10c). This anomaly was most likely caused by the misalignment of the top and root weld, where the root welding caused secondary material annealing. In this location, the hardness decreased to a value of 270 HV0.5, which is slightly lower than the minimum obtained for the SCHAZ zone (280 HV0.5). Generally, the highest values of the hardness traverse were obtained in Sample 4, where the minimal hardness reached a value of 300 HV0.5 in the SCHAZ zone. Using the MAG welding process with addition of the G3Si1 filler metal for manufacturing, Sample 5 caused a significant hardness drop in the weld metal to approx. 220 HV0.5 (Figure 10d), while the CGHAZ reaches 290 HV0.5. This indicates a significant wire undermatch.

The hardness traverse measurements also provided a possibility to approximately estimate the width of the HAZ at the measurement depth of 1 mm. It is important to note that due to the welding process character, the HAZ is not ideally symmetrical, and the results are also related to the lateral resolution of the hardness measurement (indentation distances). The results provided in Table 5 (the heat input from Table 3 was added for reference) show that the width of the HAZ was increasing from Sample 1 up to Sample 3, correlating with the increased welding heat input. Sectioning the sheet metal plates for the welding of Sample 4 did not result in a change in the width of the HAZ as it is almost identical to Sample 2, which was welded with similar heat input. Sample 5 was welded with the use of additional filler metal and a heat input higher than Sample 2, and despite the higher heat input, the weld HAZ was narrower.

## 4. Discussion

The primary aim of the study was to observe the influence of heat cycle parameters and part fixation on residual stress generated in welds manufactured from S960MC steel by TIG and MAG methods. As can be seen from the results, the welded joints exhibit different trends in the axial and tangential directions. In the axial direction, preferably tensile residual stresses are created with a maximum in the SCHAZ zone, while in the tangential direction, compressive residual stresses are created in this zone, and maximal tensile residual stresses are in the middle of the HAZ. The magnitudes of the axial residual stresses in the HAZ reach values close to 80% of the yield point and can be considered severe, since the magnitudes are on the edge of becoming an elasto-plastic deformation. Typically, in works related to residual stresses of the S960 MC butt welds, the magnitudes in the axial direction reach values close to 650 MPa [26,33,34]. However, all these works were related to application of progressive welding technologies, such as electron beam or laser welding, which operate at much lower heat inputs (approx. 1 kJ/cm). In the case of TIG autogenous welding, the residual stresses with magnitudes close to 900 MPa were also obtained by Sun et al. [18], where S960MC steel with 8 mm sheet thickness was welded with heat input of 20 kJ/cm; therefore, such high values are not uncommon for this welding technology.

Based on the obtained axial residual stress results evaluated on the surface of the autogenously welded samples, Sample 1 (2.9 kJ/cm) and Sample 2 (3.6 kJ/cm), it appears that generally the magnitude of residual stress increases with increasing heat input when using identical weld configuration. The reason for this behavior lies primarily in softening of the SCHAZ zone. As this zone is softer than the adjacent high-strength region, the material yields, while the surrounding material is mostly elastic. The plastically shortened SCHAZ is later elastically “pulled back” by the stronger material as the joint equilibrates, leaving it in the axial tension [35]. The more heat that is introduced in the material, the higher the thermal expansion, causing generation of higher residual stresses. This is also related to the cumulative heat input (Table 3), which is an integration of the delivered heat to the material with respect to the welding length—changes in the residual stress profiles might vary when shorter or longer welding lengths are considered. However, when changes in the weld sequencing and configuration are introduced, as in the case of Samples 3, 4 and 5, the residual stress generation does not follow this simple relationship, due to additional factors, such as the secondary annealing of Sample 3 by root filling welding, removing of material constraining (Samples 4 and 5) or adding filler material (Sample 5).

The reason why in tangential direction the tensile residual stresses achieved the highest magnitude in the middle of the HAZ is related to the fact that during the welding the material in the hot zone (material volume with temperature above 450 °C) has restrained ability for relative motion by the cold zone (material with temperature below 450 °C, where the thermal expansion is negligible) [16]. This leads to transversal curvature along the weld causing tension in the weld’s middle. This generates high tensile residual stresses in the HAZ due to following thermal shrinking of the weld metal. Since a part of the thermal expansion was already accommodated by the hot material plastic flow, the subsequent restrained contraction leaves tensile residual stress to satisfy the overall equilibrium. This effect is even more pronounced when the welded components are also mechanically restricted from any possible tangential relative motion, as the restrictions add to the significance of the cold zone effect. The observation agrees with work of Park et al. [36] who conclude that fixation of the welded components has significant influence, mainly on the tangential residual stresses magnitude, while the axial stresses are not sensitive to this change.

Unalike the thermal expansion, the influence of volume change during the solid-state phase transformation is not present in the whole temperature interval, as it occurs between 700 and 900 °C. During this interval, the ferrite/austenite transformation adds (heating cycle) or subducts (cooling cycle) an additional ≈1% of volume difference [37]. However, as the phase transformation occurs in the material at high temperatures, when the yield point of the material is lower, the volume changes are mostly accommodated by the plastic flow of the hot zone. This contributes to the yielding caused by thermal expansion and compels reaction forces in the HAZ.

On the other hand, the in-dept residual stress measurements are not showing such a simple dependance between the residual stress magnitude and welding heat input. The in-depth axial residual stress magnitudes of Sample 1 are higher when compared to almost all the other tested samples, mainly in the middle and end measurement locations. In the tangential direction, the residual stress magnitudes of Sample 1 differ from the surface measurement. For this case, the stresses are not as low as they were on the surface and lay on a similar level to other evaluated welds with higher heat inputs. This emphasizes the complexity of the reaction forces created during the heating cycle and influence of the surface to core stress gradients. This implies that only surface residual stress evaluation does not provide sufficient information on the real residual stress state of the HSLA welds.

Similar behavior of the heat input influence on surface residual stresses was observed by Nitschke-Pagel and Wohlfahrt in [38]. In the study, they demonstrated experimental results of welded steel by a TIG method with use of 6 kJ/cm and 20 kJ/cm heat inputs. The obtained results indicated that the heat input influences not only the magnitude, but also the distribution of axial residual stress: low heat inputs generate more compressive residual stresses and high heat inputs support generation of tensile residual stresses. In addition to the residual stress profiles, the authors also recorded the cooling curves and plotted them against the corresponding continuous cooling transformation (CCT) diagram of the used steel. The results show that selection of a lower heat input led to a lower transformation temperature, which reduced the scope of contraction stresses generated after the transformation was exhausted. This was one of the main reasons for the change in the character of residual stress. On the other hand, work of Jiang et al. [21] concludes that higher heat input during the welding process does not necessarily mean higher residual stresses, mainly in the tangential direction, and can even cause their decrease. The authors claim that the reason for this behavior is related to an increase in the welding temperature due to an increase in the heat input. When the welding temperature increases, the deformation increases, causing release of more residual stress. Therefore, the transverse stress magnitudes are decreased. This would explain the decrease in residual stress on the surface of Sample 5, visible in Figure 4, Figure 5 and Figure 6, and the variation of tangential residual stresses in the other samples. The values of residual stress in Sample 5 might be also influenced by low hardness of the filler metal, which was able to accumulate more plastic deformation caused by the thermal dilatation.

The influence of the measurement location with respect to the beginning, middle and end of the welded joint showed a difference only for the axial residual stresses, which tend to be generally higher in the middle. This applies for both surface and in-depth results. Similar behavior was also observed in [26] and is most likely related to the fact that the material at the beginning and end is closer to the edges, which are less constrained in the ability of the elasto-plastic material flow, mainly in the axial direction. However, as this behavior was still not widely studied, confirmation by future analyses might be required, and the influence of the actual temperature of the welded components, which gradually changes due to the welding, also needs to be investigated.

Besides the highest magnitude of axial residual stresses, the SCHAZ zone is also characteristic by significant reduction of mechanical properties, which is recorded by the hardness drop. This area is often referred as a “soft zone”, and for the welds in this study, the hardness drop was in the range of 13% to 18%. According to work by Amraei et al. [12], the hardness drop in the soft zone in high strength steels can reach even 60%. According to [14,39], the SCHAZ zone represents a material volume heated below the Ac_1_ temperature (less than 550 °C), where no ferrite/austenite phase transformation is present. Therefore, the grain recovery and decrease in dislocation density are the primary mechanisms responsible for the hardness drop. This was also confirmed in [40], where TEM analysis revealed that the microstructure of this zone consists of a fine martensite and bainite mixture, meaning it is identical as in the base metal. Mičian et al. [9] analyzed the relation between welding parameters and softening in high strength steels welded by the MAG method. The results of the tensile tests showed that despite the welding parameters used, each specimen fractured in the SCHAZ sub-zone, identifying it as the most critical zone for quasi-static loading. This zone also appears to be the most sensitive to cyclic loading as lower hardness causes higher yielding of the material in this zone, creating a location for localization of a cyclic elasto-plastic deformation. However, since the residual stresses tend to add to the applied forces [41,42], in the case of fatigue loading in the tangential direction, the sum of the stresses may shift the location of the fatigue crack initiation to the HAZ.

## 5. Conclusions

The aim of the study was to analyze the influence of welding cycle parameters, heat input and weld configuration on residual stress distribution in S960MC steel welded joints. Based on the obtained experimental data and critical discussion, the followng can be concluded:

- Using TIG and MAG welding technology can generate tensile residual stresses with magnitudes of 80% of the material’s yield point. The highest magnitudes present in the SCHAZ subzone are oriented in the axial direction—the direction of the welding. The SCHAZ subzone is also characterized by a significant drop caused by recovery processes in the material due to annealing at temperatures below the Ac_1_. The combination of these two factors is crucial for mechanical strength and fatigue resistance of the welded joint; however, the contribution of the axial residual stresses would be notable, mainly in the case of axial loading.
- Restraining the relative motion of the welded components results in generating high magnitudes of tensile residual stresses in the tangential direction localized in the middle of the HAZ. Although, the HAZ is not characterized by significant softening, in the case of tangential loading, the residual stresses will contribute to the total loading and might cause a shift in the initial failure location.
- With respect to the surface residual stresses, a direct relation between increasing heat input and residual stress magnitudes is observed. However, the in-depth residual stresses (measured at a depth of 0.2 mm) do not confirm this simple relationship. In the case of the lowest applied heat input for TIG autogenous welding, the surface residual stresses showed significantly lower values when compared to the in-depth measurements. This indicates that evaluating only surface residual stresses can provide false assumptions of the weld’s residual stress state.
- Evaluation of surface residual stresses in the axial direction at the beginning of the weld shows the highest scatter among all the measured locations. However, such a notable scatter was not observed in either the tangential direction or in the in-depth measurements. With respect to the weld location, without few exceptions (e.g., Sample 2), the highest axial residual stresses were typically present in the middle, indicating a possible influence of the edge proximity and immediate material temperature. Nevertheless, the results are not conclusive and would require further investigation.

## Figures and Tables

**Table 1:** Chemical composition of S960MC per inspection certificate (EN 10204 3.1a).

Chemical Composition [wt. %]												
C	Mn	Si	P	S	Cr	Mo	Ni	Nb	Ti	V	Al	Fe
0.079	1.791	0.282	0.003	0.003	0.210	0.269	0.018	0.005	0.009	0.030	0.055	Balance

**Table 2:** Mechanical properties of S960MC.

Yield Point R_p0.2_ [MPa]	Ultimate Tensile Strength R_m_ [MPa]	Ductility A [%]
1095	1160	7

**Table 3:** Welding parameters for sample manufacturing.

Sample No.	Welding Voltage U_W_ [V]	Welding Current I_W_ [A]	Welding Speed V_W_ [cm/s]	Heat Input Q [kJ/cm]	Cumulative Heat Input [kJ]	Welding Method	Shielding Gas	Filler Metal
1	12.3	135	0.35	2.9	40.6	TIG	Ar	none
2	12.3	170	0.35	3.6	50.4	TIG	Ar	none
3—surface	12.6	170	0.35	3.7	51.8	TIG	Ar	none
3—root	12.3	125	0.35	2.6	36.4	TIG	Ar	none
4	12.6	175	0.35	3.8	57	TIG	Ar	none
5	12.3	161	0.5	4.8	72	MAG	82% Ar + 18% CO_2_	G3Si1

**Table 4:** The reference value of the residual stresses in the as-received material.

	Surface		Depth 0.2 mm	
	Axial	Tangential	Axial	Tangential
Residual stress [MPa]	−61	−183	−32	−93

**Table 5:** Width of the HAZ.

Sample No.	Width [mm]	Heat Input Q [kJ/cm]
1	11.2	2.9
2	12.8	3.6
3	13.8	3.7 + 2.6
4	12.9	3.8
5	11.6	4.8

## Data Availability

The data that supports the findings of this study are available on request from the corresponding author, L.T.

## Abbreviations

The following abbreviations are used in this manuscript:

TIG	Tungsten Inert Gas welding
MAG	Metal Active Gas welding
HSLA	high strength low alloy steels
QT	quenching and tempering
TMCP	thermo-mechanical control processing
HAZ	heat affected zone
WM	weld metal
UTS	Ultimate Tensile Strength
FZ	Fusion zone
CGHAZ	Coarse-Grained Heat-Affected Zone
FGHAZ	Fine-Grained Heat Affected Zone
ICHAZ	Inter-Critical Heat Affected Zone
SCHAZ	Sub-Critical Heat Affected Zone
CCT	continuous cooling transformation

## Appendix A

### Appendix A.1

**Table A1:** Welding residuals stresses–Sample 1, surface.

Axial Direction														
Location	SCHAZ	Std. dev.	SCHAZ/HAZ	Std. dev.	HAZ	Std. dev.	FZ/WM	Std. dev.	HAZ	Std. dev.	HAZ/SCHAZ	Std. dev.	SCHAZ	Std. dev.
Beginning	148	5.5	60	12	83	6.7	147	34	161	19.6	−31	5.9	46	10.3
Middle	133	12.8	−12	9.3	138	16.2	228	26.8	218	34.7	−7	6.6	63	9.3
End	143	13	34	15.8	135	13.1	172	27.4	287	14.2	45	7	48	10.9
Tangential direction														
Beginning	−183	7.7	−204	9.3	82	16.1	140	28	97	20.5	−127	10.4	−257	10.7
Middle	−120	10.7	−222	10.9	140	21	164	16.8	93	23	−130	9	−143	8.3
End	−62	5.4	−115	10.6	295	20.6	212	14.2	307	25	−68	12.7	−183	5.2

**Table A2:** Welding residuals stresses—Sample 1, at a depth of 0.2 mm.

Axial Direction														
Location	SCHAZ	Std. dev.	SCHAZ/HAZ	Std. dev.	HAZ	Std. dev.	FZ/WM	Std. dev.	HAZ	Std. dev.	HAZ/SCHAZ	Std. dev.	SCHAZ	Std. dev.
Beginning	560	7.9	369	11.4	57	23.1	144	45.9	53	9.5	431	11.6	479	9.5
Middle	815	9.5	421	11.9	271	13.2	339	16.5	349	18.9	321	11	795	10.2
End	858	8.4	337	10	165	7.8	131	20	202	68.9	144	7.3	837	9.5
Tangential direction														
Beginning	−148	28.3	−266	27.6	−32	24.1	15	39.4	29	20.5	−197	31.5	−170	23.4
Middle	38	25.3	−212	27.1	168	25.1	162	21.3	281	39.7	−145	24.7	−67	26.9
End	37	24.6	−78	24.9	272	23.2	111	16.6	335	23.7	7	27.3	−2.2	24.5

### Appendix A.2

**Table A3:** Welding residuals stresses—Sample 2, surface.

Axial Direction														
Location	SCHAZ	Std. dev.	SCHAZ/HAZ	Std. dev.	HAZ	Std. dev.	FZ/WM	Std. dev.	HAZ	Std. dev.	HAZ/SCHAZ	Std. dev.	SCHAZ	Std. dev.
Beginning	526	5.4	379	5.7	284	10.1	137	64.1	237	28.7	178	10.2	561	4.5
Middle	556	11.4	232	7.7	397	9.4	383	29.9	476	14.8	197	6.6	523	10.9
End	357	8.8	403	9.7	409	12.6	178	36.8	454	15	240	6.4	576	8.7
Tangential direction														
Beginning	−131	15.8	−152	14.2	362	11.1	34	31.4	355	36	−166	16.6	−150	13.7
Middle	−15	7.4	−43	8.5	475	14.4	302	17.1	546	14.5	43	8.4	−33	16.6
End	−57	12.3	111	11.5	720	20.6	76	30.8	804	20.3	104	11	−35	8

**Table A4:** Welding residuals stresses—Sample 2, at a depth of 0.2 mm.

Axial Direction														
Location	SCHAZ	Std. dev.	SCHAZ/HAZ	Std. dev.	HAZ	Std. dev.	FZ/WM	Std. dev.	HAZ	Std. dev.	HAZ/SCHAZ	Std. dev.	SCHAZ	Std. dev.
Beginning	423	4.6	467	6.2	28	6.7	106	36.7	34	26.9	118	5.6	557	7.3
Middle	765	9.1	300	11	339	18.6	301	31	353	11.5	294	9.5	729	8.5
End	797	6.2	285	7.6	144	27.2	200	41	129	22.5	78	6.3	670	4.3
Tangential direction														
Beginning	−203	35.7	−280	43.9	−40	24.3	−120	37.3	71	26.1	−277	49	−246	40.9
Middle	−78	37.6	44	31	477	22.3	−85	22.4	367	21.2	−56	46.9	−18	36.7
End	−100	40.9	−47	49.7	244	22.5	−50	32.7	315	29.5	−14	51.6	−127	35.6

### Appendix A.3

**Table A5:** Welding residuals stresses—Sample 3, surface.

Axial Direction														
Location	SCHAZ	Std. dev.	SCHAZ/HAZ	Std. dev.	HAZ	Std. dev.	FZ/WM	Std. dev.	HAZ	Std. dev.	HAZ/SCHAZ	Std. dev.	SCHAZ	Std. dev.
Beginning	57	11	87	16.1	240	9.3	154	27.7	417	12.7	224	18.2	425	18.5
Middle	617	15.7	313	11.2	404	9.3	227	14.1	563	11.6	342	14.3	624	23.3
End	572	17.7	304	18.3	377	10.8	122	35.1	524	17.4	209	11.2	495	13.7
Tangential direction														
Beginning	−169	7.5	−151	5	158	12	69	26.6	580	18.6	52	6.7	−151	6.2
Middle	69	4.3	33	5.3	492	11.8	155	33.3	742	21.6	219	5.5	39	6.1
End	−92	7.4	40	11.2	680	23.4	169	22.4	777	18.6	151	6.8	−78	4.8

**Table A6:** Welding residuals stresses—Sample 3, at a depth of 0.2 mm.

Axial Direction														
Location	SCHAZ	Std. dev.	SCHAZ/HAZ	Std. dev.	HAZ	Std. dev.	FZ/WM	Std. dev.	HAZ	Std. dev.	HAZ/SCHAZ	Std. dev.	SCHAZ	Std. dev.
Beginning	536	39.9	383	42.6	46	24	130	28.3	120	22.1	83	40.3	570	51.6
Middle	906	41	413	50.3	276	24.4	387	34.9	380	20.8	259	36.9	790	56
End	697	52.5	303	45.8	65	91.9	85	59.2	119	27.2	19	42.1	633	48.6
Tangential direction														
Beginning	−139	7.2	−207	7.8	24	10.3	−70	44.3	292	27.6	−141	5.4	−173	2.7
Middle	68	10.2	29	6.4	280	13.5	28	37.3	534	19.3	62	5.9	27	6.5
End	−142	4.9	−135	9.5	149	24.4	−80	38.3	289	19.6	−119	6	−168	8.2

### Appendix A.4

**Table A7:** Welding residuals stresses—Sample 4, surface.

Axial Direction														
Location	SCHAZ	Std. dev.	SCHAZ/HAZ	Std. dev.	HAZ	Std. dev.	FZ/WM	Std. dev.	HAZ	Std. dev.	HAZ/SCHAZ	Std. dev.	SCHAZ	Std. dev.
Beginning	371	8.3	172	10.1	271	11	87	49.3	380	33.9	117	5.7	368	8
Middle	654	11.2	348	9.9	412	10.2	251	35.4	563	37.1	346	14.3	504	9.9
End	420	10.7	167	6.7	163	11.8	333	44	421	17.4	310	10.8	419	9
Tangential direction														
Beginning	−161	9.3	−154	5	302	9	139	46.8	407	22	6	7.6	−155	3.9
Middle	45	5.4	−64	5.2	344	12.1	−40	25.1	649	49.1	202	10.1	−82	6.9
End	−153	4.9	−137	6.6	188	11.3	436	31.7	752	35.8	348	11.4	−108	4.5

**Table A8:** Welding residuals stresses—Sample 4, at a depth of 0.2 mm.

Axial direction														
Location	SCHAZ	Std. dev.	SCHAZ/HAZ	Std. dev.	HAZ	Std. dev.	FZ/WM	Std. dev.	HAZ	Std. dev.	HAZ/SCHAZ	Std. dev.	SCHAZ	Std. dev.
Beginning	426	23.3	374	25.8	−42	23.1	93	41.9	122	33.7	34	20.9	579	29.2
Middle	865	28.1	491	17	225	25	255	67	334	24.6	278	22.6	804	28.3
End	549	28.9	162	19.9	15	20.4	97	32.1	99	27.4	18	19.5	536	28.6
Tangential direction														
Beginning	−236	7.4	−239	7	93	13.7	−69	45.5	91	13.5	−211	9.3	−203	8.7
Middle	33	8.3	−84	4.5	240	11.9	−35	53.2	389	16.9	−12	7.3	−63	7.4
End	−178	8.1	−158	4.9	71	10.4	25	23	195	19	−22	8.4	−176	5.9

### Appendix A.5

**Table A9:** Welding residuals stresses—Sample 5, surface.

Axial Direction														
Location	SCHAZ	Std. dev.	SCHAZ/HAZ	Std. dev.	HAZ	Std. dev.	FZ/WM	Std. dev.	HAZ	Std. dev.	HAZ/SCHAZ	Std. dev.	SCHAZ	Std. dev.
Beginning	364	19.4	148	10.9	−16	8.8	394	41.3	283	13.8	163	13.3	503	16.2
Middle	549	16	253	11.9	125	10	403	43.1	460	12	381	18.4	739	13.8
End	747	29	567	32.9	274	20.9	318	20.9	462	10.1	360	11.8	792	19.9
Tangential direction														
Beginning	−189	4.9	−160	6.7	−74	8.8	26	40	295	8.4	−69	4.7	−210	4.6
Middle	72	5.8	31	4.9	223	6.9	−34	28.9	371	9.9	94	8.7	30	4.3
End	52	4.7	85	7	318	10.4	23	27.2	410	9.7	222	5.5	25	7.1

**Table A10:** Welding residuals stresses—Sample 5, at a depth of 0.2 mm.

Axial Direction														
Location	SCHAZ	Std. dev.	SCHAZ/HAZ	Std. dev.	HAZ	Std. dev.	FZ/WM	Std. dev.	HAZ	Std. dev.	HAZ/SCHAZ	Std. dev.	SCHAZ	Std. dev.
Beginning	469	41.9	493	47.6	111	44.9	279	15.2	225	30.5	94	20.1	586	28.3
Middle	901	37.5	740	46.8	364	46.6	427	17.8	390	23.8	403	29.2	855	31.3
End	804	50.4	465	62.8	150	50.6	301	32.7	213	20.8	326	22.4	834	30
Tangential direction														
Beginning	−214	8.6	−169	7.7	197	16.2	−116	11.6	274	20	−106	5.6	−246	6.3
Middle	98	4.9	35	5.9	161	6.7	−100	27.3	279	10.3	86	7.3	46	6.7
End	25	10.1	9	6.7	152	9.2	−77	21	405	13.7	94	6.7	6	5.6

## References

[1] Nie Y., Shang C., You Y., Li X., Cao J., He X. (2010). 960 MPa Grade High Performance Weldable Structural Steel Plate Processed by Using TMCP. J. Iron Steel Res. Int..

[2] Maraveas C., Fasoulakis Z.C., Tsavdaridis K.D. (2017). Mechanical Properties of High and Very High Steel at Elevated Temperatures and after Cooling Down. Fire Sci. Rev..

[3] Klein M., Spindler H., Luger A., Rauch R., Stiaszny P., Eigelsberger M. (2005). Thermomechanically Hot Rolled High and Ultra High Strength Steel Grades—Processing, Properties and Application. Mater. Sci. Forum.

[4] Wang Y., Li F., Li Y., Li J., Chen H., Bai Y. (2025). Effects of Ultrasonic Surface Rolling Process on Microstructure and Mechanical Properties of Metastable Fe50Mn30Co10Cr10 High-Entropy Alloy Fabricated by Laser Directed Energy Deposition. J. Mater. Res. Technol..

[5] Keränen L., Nousiainen O., Javaheri V., Kaijalainen A., Pokka A.-P., Keskitalo M., Niskanen J., Kurvinen E. (2022). Mechanical Properties of Welded Ultrahigh-Strength S960 Steel at Low and Elevated Temperatures. J. Constr. Steel Res..

[6] Tümer M., Schneider-Bröskamp C., Enzinger N. (2022). Fusion Welding of Ultra-High Strength Structural Steels—A Review. J. Manuf. Process..

[7] Schaupp T., Schroepfer D., Kromm A., Kannengiesser T. (2017). Welding Residual Stresses in 960 MPa Grade QT and TMCP High-Strength Steels. J. Manuf. Process..

[8] Guo W., Li L., Dong S., Crowther D., Thompson A. (2017). Comparison of Microstructure and Mechanical Properties of Ultra-Narrow Gap Laser and Gas-Metal-Arc Welded S960 High Strength Steel. Opt. Lasers Eng..

[9] Mičian M., Frátrik M., Kajánek D. (2021). Influence of Welding Parameters and Filler Material on the Mechanical Properties of HSLA Steel S960MC Welded Joints. Metals.

[10] St. Węglowski M., Zeman M. (2014). Prevention of Cold Cracking in Ultra-High Strength Steel Weldox 1300. Arch. Civ. Mech. Eng..

[11] Gáspár M. (2019). Effect of Welding Heat Input on Simulated HAZ Areas in S960QL High Strength Steel. Metals.

[12] Amraei M., Afkhami S., Javaheri V., Larkiola J., Skriko T., Björk T., Zhao X.-L. (2020). Mechanical Properties and Microstructural Evaluation of the Heat-Affected Zone in Ultra-High Strength Steels. Thin Walled Struct..

[13] Amraei M., Ahola A., Afkhami S., Björk T., Heidarpour A., Zhao X.-L. (2019). Effects of Heat Input on the Mechanical Properties of Butt-Welded High and Ultra-High Strength Steels. Eng. Struct..

[14] Mičian M., Frátrik M., Brůna M. (2024). Softening Effect in the Heat-Affected Zone of Laser-Welded Joints of High-Strength Low-Alloyed Steels. Weld. World.

[15] Lin Z., Song K., Sun Z., Zhu Z., Zhao X., Goulas C., Ya W., Yu X. (2024). Mechanical Performance of 22SiMn2TiB Steel Welded with Low-Transformation-Temperature Filler Wire and Stainless Steel Filler Wire. J. Iron Steel Res. Int..

[16] Sun J., Nitschke-Pagel T., Dilger K. (2023). Generation and Distribution Mechanism of Welding-Induced Residual Stresses. J. Mater. Res. Technol..

[17] Tichoň D., Vojtek T., Jambor M., Dlhý P., Trško L., Vlček L., Náhlík L., Hutař P. (2026). Fatigue Life Prediction of Weld Joints: Microstructural Variation Can Be Omitted While Residual Stress Consideration Is Essential. Eng. Fract. Mech..

[18] Sun J., Dilger K. (2023). Influence of Initial Residual Stresses on Welding Residual Stresses in Ultra-High Strength Steel S960. J. Manuf. Process..

[19] Sun J., Dilger K. (2023). Influence of Preheating on Residual Stresses in Ultra-High Strength Steel Welded Components. J. Mater. Res. Technol..

[20] Guo Q., Du B., Xu G., Chen D., Ma L., Wang D., Zhang Y. (2020). Influence of Filler Metal on Residual Stress in Multi-Pass Repair Welding of Thick P91 Steel Pipe. Int. J. Adv. Manuf. Technol..

[21] Jiang W.C., Wang B.Y., Gong J.M., Tu S.T. (2011). Finite Element Analysis of the Effect of Welding Heat Input and Layer Number on Residual Stress in Repair Welds for a Stainless Steel Clad Plate. Mater. Des..

[22] Alipooramirabad H., Paradowska A., Ghomashchi R., Reid M. (2017). Investigating the Effects of Welding Process on Residual Stresses, Microstructure and Mechanical Properties in HSLA Steel Welds. J. Manuf. Process..

[23] Alipooramirabad H., Ghomashchi R., Paradowska A., Reid M. (2016). Residual Stress- Microstructure- Mechanical Property Interrelationships in Multipass HSLA Steel Welds. J. Mater. Process. Technol..

[24] Schroepfer D., Kannengiesser T. (2016). Stress Build-up in HSLA Steel Welds Due to Material Behaviour. J. Mater. Process. Technol..

[25] Guo W., Francis J.A., Li L., Vasileiou A.N., Crowther D., Thompson A. (2016). Residual Stress Distributions in Laser and Gas-Metal-Arc Welded High-Strength Steel Plates. Mater. Sci. Technol..

[26] Sisodia R.P.S., Gáspár M., Sepsi M., Mertinger V. (2021). Comparative Evaluation of Residual Stresses in Vacuum Electron Beam Welded High Strength Steel S960QL and S960M Butt Joints. Vacuum.

[27] Riofrío P.G., Antunes F., Ferreira J., Batista A.C., Capela C. (2021). Fatigue Performance of Thin Laser Butt Welds in HSLA Steel. Metals.

[28] Molina-Castillo A.E., López-Baltazar E.A., Alvarado-Hernández F., Gómez-Jiménez S., Espinosa-Lumbreras J.R., Ruiz Mondragón J.J., Baltazar-Hernández V.H. (2025). Effect of the Heat Affected Zone Hardness Reduction on the Tensile Properties of GMAW Press Hardening Automotive Steel. Metals.

[29] Nishimura R., Ma N., Liu Y., Li W., Yasuki T. (2021). Measurement and Analysis of Welding Deformation and Residual Stress in CMT Welded Lap Joints of 1180 MPa Steel Sheets. J. Manuf. Process..

[30] Wang L., Qian X., Feng L. (2024). Effect of Welding Residual Stresses on the Fatigue Life Assessment of Welded Connections. Int. J. Fatigue.

[31] Wang L., Qian X. (2022). Welding Residual Stresses and Their Relaxation under Cyclic Loading in Welded S550 Steel Plates. Int. J. Fatigue.

[32] (2023). Metallic Materials—Vickers Hardness Test.

[33] Mičian M., Frátrik M., Moravec J., Jambor M., Solfronk P., Šulák I. (2026). Enhancement of Fatigue Performance of Thin HSLA Steel Laser-Welded Butt Joints. Weld. World.

[34] Sisodia R.P.S., Gigli L., Plaisier J., Mertinger V., Weglowski M.S., Sliwinski P. (2024). Synchrotron Diffraction Residual Stresses Studies of Electron Beam Welded High Strength Structural Steels. J. Mater. Res. Technol..

[35] Guzman J., Riffel K.C., McDonnell M., Bunn J., Payzant A., Kyle D., Ramirez A.J. (2026). Correlation between Microstructure and Residual Stress Formation in Friction Stir Welded Armor Steels Characterized by Neutron Diffraction. J. Mater. Process. Technol..

[36] Park J., An G., Ma N., Kim S.-J. (2023). Prediction of Transverse Welding Residual Stress Considering Transverse and Bending Constraints in Butt Welding. J. Manuf. Process..

[37] Lv S., Wu H.-H., Wang K., Zhu J., Wang S., Wu G., Gao J., Yang X.-S., Mao X. (2023). The Austenite to Polygonal Ferrite Transformation in Low-Alloy Steel: Multi-Phase-Field Simulation. J. Mater. Res. Technol..

[38] Nitschke-Pagel T., Wohlfahrt H. (2002). Residual Stresses in Welded Joints—Sources and Consequences. Mater. Sci. Forum.

[39] Zhang X., Li C., Yang X., Di X. (2024). Effect of Quenching-and-Tempering Heat Treatment on Mechanical Properties and Heat-Affected Zone Softening Behavior of Ultra-High Strength Steel. J. Mater. Eng. Perform..

[40] Strakova D., Jambor M., Novy F., Trsko L. (2025). Microstructure Evolution in the Heat Affected Zone of the S960MC Weld Joint. Int. J. Adv. Manuf. Technol..

[41] Chiocca A., Frendo F., Aiello F., Bertini L. (2022). Influence of Residual Stresses on the Fatigue Life of Welded Joints. Numerical Simulation and Experimental Tests. Int. J. Fatigue.

[42] Harati E., Karlsson L., Svensson L.-E., Dalaei K. (2015). The Relative Effects of Residual Stresses and Weld Toe Geometry on Fatigue Life of Weldments. Int. J. Fatigue.

